# Comprehensive Sonographic Paradigm and Trend Pattern of Median Nerve Indices in Carpal Tunnel Syndrome from Wrist to Forearm: What We Need to Know

**DOI:** 10.3390/diagnostics16040641

**Published:** 2026-02-23

**Authors:** Adeena Khan, Fawaz T. Salamah, Syed S. Habib, Waleed Fawzy, Fawzia AlRouq, Huthayfah T. Alkhliwi, Mamoona Sultan, Ahmed O. Alsabih

**Affiliations:** 1 Department of Radiology and Medical Imaging, King Saud University, Riyadh 11451, Saudi Arabia; 2 Department of Physiology, King Saud University, College of Medicine, Riyadh 11451, Saudi Arabia; 3 Department of Internal Medicine, King Saud University, Riyadh 11451, Saudi Arabia

**Keywords:** carpal tunnel syndrome, ultrasound, median nerve, cross-sectional area, proximal to inlet, inlet, forearm

## Abstract

**Objective:** The study aim was panoramic sonographic inspection of the median nerve (MN) from the wrist to the forearm in cases and controls. Additionally, integration of comparisons at various levels may aid in identifying principal ultrasound parameters of carpal tunnel syndrome (CTS). **Methods:** Dynamic, static, and panoramic sonographies of 65 healthy and 83 CTS hands were performed. Multileveled qualitative (MN and flexor retinaculum morphology) and quantitative (cross-sectional area CSA, differences, and ratios) MN variables for CTS, followed by comparative statistical analysis to predict values and patterns of MN, were derived. **Results:** Subjectively, hypoechoic, vascular, compressed, hypomobile MN and bowed thickened flexor retinaculum were significantly more prevalent in cases (28.9–66.3%) than in controls (0–7.7%). Objectively, the proximal to inlet (pi) and the forearm at 12 cm (12) were the most representative sites. The area under curve (AUC) values for the MN dimensions, in decreasing order, were 0.9, 0.89, 0.86, and ≤0.80 for the CSA difference ‘pi’ and ‘12’ (Cpi-C12), the CSA proximal to inlet (Cpi), the ratio of CSA at pi and 12 (Cpi/C12), and the CSA at inlet (Ci), respectively. Their cut-off values were 3.7, 9.1, 1.8, and 7.2 mm^2^, respectively. Differences and ratios between ‘Cpi’ and ‘Ci’ were less reliable (AUC ≤ 0.74, sensitivity ≤ 61.4%). Flexor retinaculum bowing, thickening, and MN flattening ratios were unreliable. **Conclusions:** Sensitivity, specificity, and precision of MN sonographic parameters in CTS increase by utilizing differences and ratios between wrist and forearm rather than isolated values. The recommended site in wrist is proximal to the inlet, and in the forearm, the best site to determine ratios and differences is at 12 cm from the distal wrist crease.

## 1. Introduction

CTS is the most common compressive MN neuropathy. Its overall lifetime prevalence is 8% and is twice as high in females as in males (10% compared to 5.8%) [1]. Causative factors of MN entrapment can be physiological, anatomical, systemic, or space-occupying lesions. However, the idiopathic variety is most frequent and found in 50% of cases [2].

Despite various diagnostic tools with predominance of nerve conduction studies (NCS), ultrasound (US) has a promising role to detect early changes in a diseased nerve with comparable sensitivity (sn) and specificity (sp) varying between 57 and 98%, average diagnostic accuracy of 82.2%, and a lower false positive rate (23%) in asymptomatic individuals. It has an added advantage to diagnose primary and secondary regional etiologies of MN entrapment with the nascent role of shear wave elastography, which is a limitation of NCS [1,2,3,4,5]. NCS is reported to show 10% and 15% false negative and false positive rates, respectively. About 16–34% of clinically defined cases of CTS are prone to being missed on NCS, but in equivocal and surgical cases, it is considered the gold standard [2,3,6,7]. There is also a significant difference in the incidence of symptomatic CTS versus (ǁ) NCS positive CTS patients (93 ǁ 40 per 1000 persons/year) [1]. The above data support the requirement for a better and less invasive diagnostic investigation with identification of cardinal variables that confirm CTS, and ultrasound has the potential to achieve this.

A literature review reveals that various threshold values of US parameters have been ascribed to swollen MN; however, these remain a subject of debate [8,9]. This could be due to the selection of various US methods, the variation in physical characteristics of samples, and the subjective selection for the best sensitivity and specificity pairs. Interobserver variation also contributes to the lack of consensus [10,11]. These contradictions are, in turn, prone to diagnostic and treatment pitfalls. Moreover, comprehensive data about MN in the wrist and forearm within a single dedicated study remains scarce; hence, a dataset that simultaneously compares multidirectional particulars of MN in CTS is unavailable. Collective description of MN variables is only found in review articles, which lack analytical connotation [2,12,13]. It is also observed that a limited number of studies have major analytical input from radiologists with a deficient description about unified, detailed, and practical CTS parameters [6,7,14,15,16].

In the literature, parameters of the MN from distal tunnel outlet in the wrist to 12 cm in forearm have been described sonographically. The nerve in the forearm has been studied for ratio and difference calculations; however, unlike our research, previous studies chose to study the MN in the forearm at only one level at a time. High-quality resolution of MN in the distal wrist is not achievable in every setting; hence, it is not suggested as a favorable site for quoting reference values and recommendations. This led us to obtain measurements in the proximal wrist only [8,17,18,19,20]. A comprehensive description of the trend of all MN dimensions from wrist to forearm in both diseased and healthy subjects also needs to be elucidated and is part of our manuscript. Sonographic parameters could be helpful in deciding between surgical interventions and less invasive procedures of nerve release and here US plays a significant role [21]. Identification of the length of nerve entrapment in our data might be a useful addition for surgical planning and a new severity assessment in CTS. Our research has documented all attainable quantitative and qualitative MN sonographic parameters, with an additional merit of comparing them with both internal and external controls. Newly introduced parameters, such as ratios and differences at various levels of the forearm, are also an idiosyncrasy of this article, and were included to avoid selection bias. They could help clinicians make precise decisions between medical and surgical management of CTS.

## 2. Materials and Methods

### 2.1. Patient Selection

**Figure 1:**
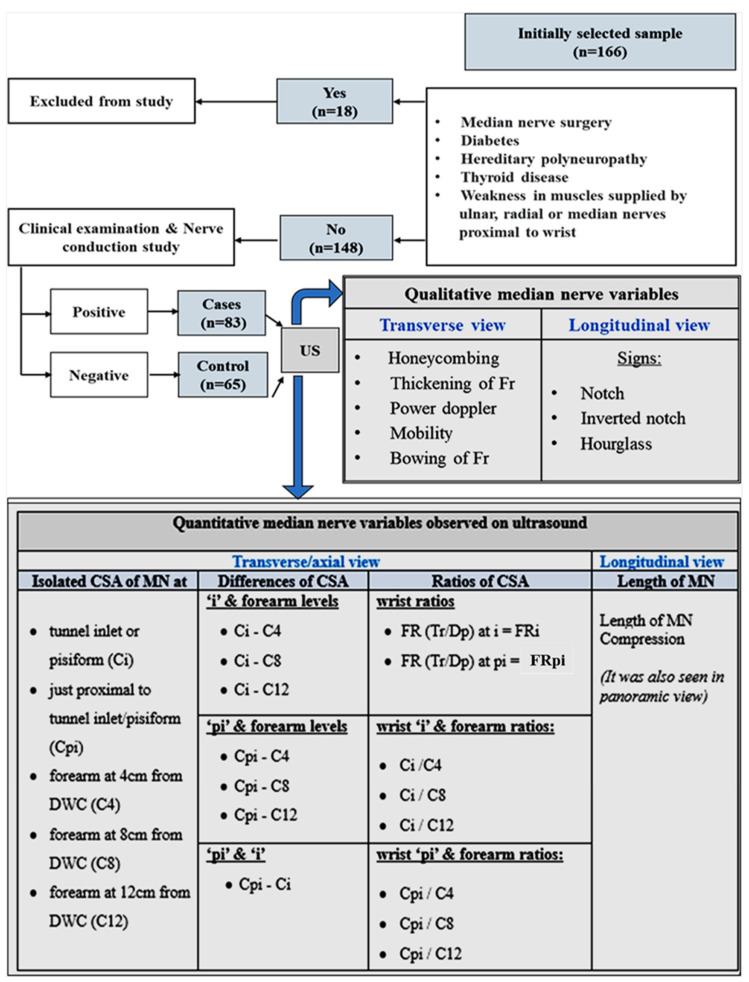
A flowchart and tabulated summary of patient selection and the imaging parameters recorded in our study. *US = ultrasound, Fr = flexor retinaculum, C or CSA = cross-sectional area, MN = median nerve, DWC = distal wrist crease, FR = flattening ratio, Tr = transverse, Dp = depth, i = inlet, pi = proximal to inlet.*

The study protocol was approved by our university institutional review board (Project No. E-21-5967). After approval, a total of 166 unilateral or bilateral hand samples were incorporated into this prospective study. Consent was obtained from all patients using a drafted data collection form. Patients were fully anonymized with respect to their sonographic images and identification data. Patients who were clinically and NCS positive, fulfilling the inclusion and exclusion criteria, were referred from the Primary Clinic to the Radiology and Imaging Department between 13 March 2022 and 12 February 2023 for US evaluation of the MN. By the study’s definition of inclusion criteria, clinically positive subjects including adults (>18 years) with numbness or tingling of the lateral three fingers triggered by manual activities and relieved by shaking the hand, hand numbness or tingling that awoke the patient from sleep at least once in two weeks, or a positive Tinel’s sign or Phalen’s test were selected for US. Patient selection is further illustrated in Figure 1.

### 2.2. Ultrasound Technique

Before performing ultrasound, the forearms of patients were marked at 4, 8 and 12 cm from distal wrist crease (DWC). A GE LOGIQ E9 (General Electric Health care, Riyadh, Saudi Arabia) ultrasound machine with a superficial probe of frequency 9–15 MHz was used for the examination. Patients were seated on a couch with the hand supine, resting on a pillow, the elbow flexed, and the fingers in a neutral or semi-extended position. Static as well as video images were saved according to the required US parameters and sent to the picture archiving and communication system for image interpretation.

### 2.3. Images Recording and Interpretation

A single senior radiologist (>10 years of musculoskeletal imaging experience), blinded to the NCS result and clinical history, performed the US. Images were interpreted by two radiologists experienced in the field of MN ultrasound, with Intraclass Correlation Coefficients (ICC) or Cohen’s Kappa values >0.80. Considering the varying anatomy of the MN at each step, we defined ‘i’ and ‘pi’ meticulously, taking DWC, probe marker line (PML) and probe upper border (PUB) as references.

#### 2.3.1. Definition of Inlet (i)

If the PML is anatomically placed at the DWC, it corresponds to the pisiform/scaphoid level, which is the site of the proximal end of the flexor retinaculum (Fr), and is hence labelled as ‘i’.

#### 2.3.2. Definition of Proximal to Inlet (pi)

If the PML is placed at the proximal wrist crease or the PUB is along the DWC, it represents the area just proximal to ‘i’ or the upper margin of Fr or pisiform/scaphoid, and is hence labelled as ‘pi’.

**Figure 2:**
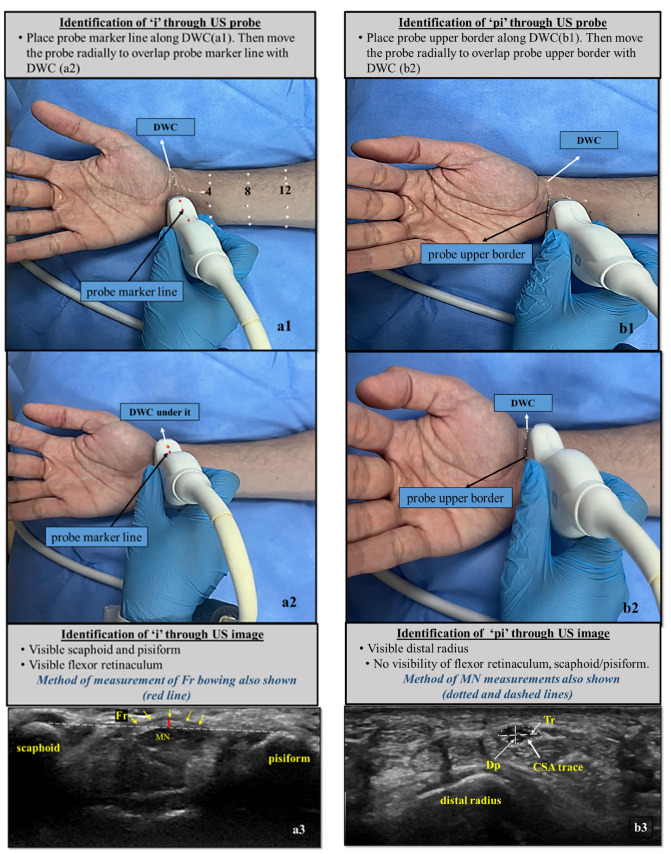
Pictures of wrist with reference markings of the sites where ultrasound examination of median nerve was performed, i.e., at ‘i’, ‘pi’, 4,8,12 cm from DWC (**a1**–**b2**). Corresponding sonographic appearance of MN at ‘i’ and ‘pi’, method of MN measurements taken and identification of location are also shown (**a3**,**b3**). Multiple downward arrows = flexor retinaculum, dashed horizontal line in (**a3**) = reference line drawn between scaphoid and pisiform to measure bowing (vertical red line) of Fr, dashed horizontal line in (**b3**) = Tr dimension of MN, dashed vertical line in (**b3**) = Dp of MN, dotted circle in (**b3**) = CSA of MN. *US = ultrasound, DWC = distal wrist crease, Fr = flexor retinaculum, CSA = cross-sectional area, Tr = transverse, Dp = depth, carpal tunnel inlet = ’i’, proximal to carpal tunnel inlet ‘pi’, MN = median nerve.*

US was started in the axial position by putting the probe perpendicular to the nerve to avoid anisotropy artifacts. The initially analyzed locations were the wrist at ‘i’ and ‘pi’. Gray scale imaging was followed by power Doppler analysis. In axial views, CSA, transverse (Tr) and depth (Dp) of MN at the marked levels of wrist (‘i’ and ‘pi’) were taken (Figure 2).

**Figure 3:**
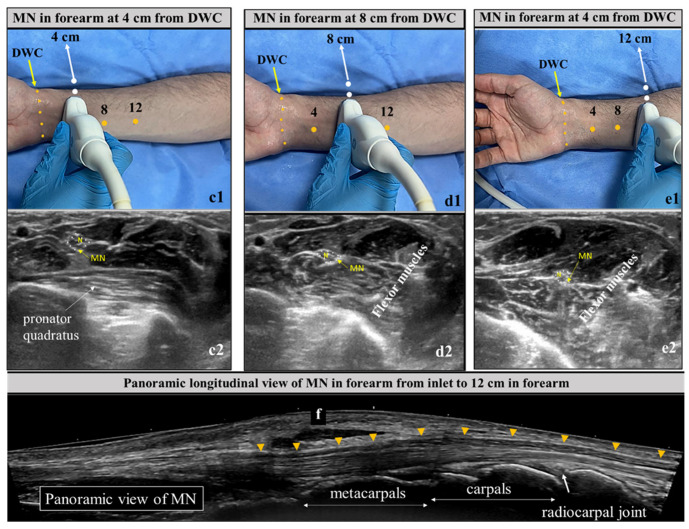
Pictures of wrist and forearm with reference markings of the sites at 4, 8 and 12 cm from DWC. Probe marker line (dotted white) is aligned with the markings to examine MN (**c1**,**d1**,**e1**). Corresponding transverse US appearance of MN at these points also shown. MN at 4 cm and 8, 12 cm are identified by pronator quadratus and flexor muscles, respectively (**c2**,**d2**,**e2**). Panoramic view longitudinal of the course of MN (arrowheads) from wrist to distal forearm also shown (**f**). *US = ultrasound, DWC = distal wrist crease, MN = median nerve.*

CSA of the forearm MN was measured at 4, 8 and 12 cm (C4, C8, C12) (Figure 3) from the DWC. Video recordings were subjectively analyzed for nerve mobility, while the rest of the parameters were checked on static images. Mobility was assessed on both axial and longitudinal views for the final impression.

Longitudinal and panoramic views were used to evaluate the length of nerve compression, to assess the notch sign, and to confirm findings recorded in axial images to reduce sampling error. Ratios and differences were calculated during data analysis. All CTS parameters (qualitative and quantitative) recorded in our study are tabulated in Figure 1.

### 2.4. Statistical Analysis

Statistical Package for Social Sciences (SPSS Version 19, Chicago, IL, USA) was used for data analysis. Descriptive characteristics were expressed as mean ± standard deviation (SD) for quantitative variables and as proportions for qualitative data. Group comparisons were performed using *t*-tests for quantitative variables and chi-square for proportions. A *p*-value < 0.05 was considered significant. Participants were dichotomized into control and CTS cases to use this as the state variable for receiver operating curve analysis, which was carried out to assess the predictive value of different MN indices (cross-sectional area, transverse length, depth, differences of CSA and ratios) as diagnostic indicators of CTS.

## 3. Results

### 3.1. Subject Characteristics and Frequency Distribution of Qualitative Data Variables

The majority of case and control subjects in our study were females (80.7% and 70.7%, respectively) and had a mean age between 40 and 50 years. Demographic characteristics are tabulated in detail (Table 1).

**Figure 4:**
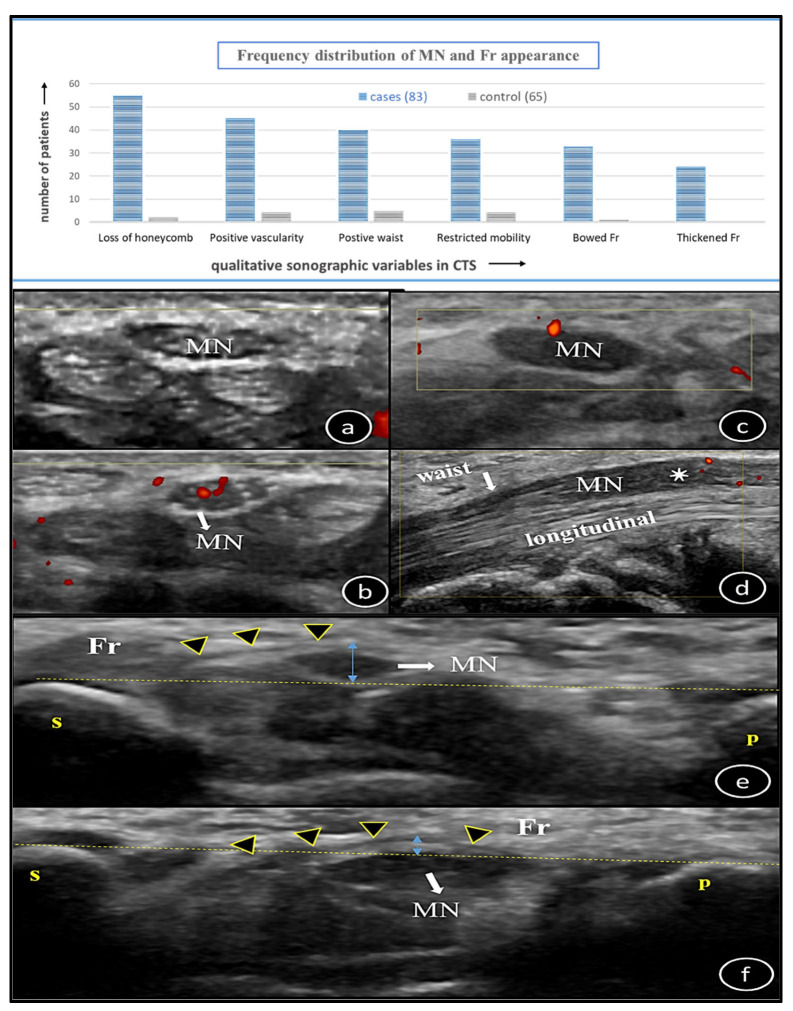
Bar graphical representation of qualitative variables recorded in CTS and control subjects in decreasing order. Below the graph is the illustration of these variables on ultrasound (**a**–**f**). Axial views (**a**–**c**): Normal nerve honeycomb appearance without vascularity (**a**), normal nerve honeycomb appearance with vascularity (orange dots) (**b**), swollen nerve with loss of honeycomb appearance and positive vascularity (**c**). Longitudinal view (**d**): Swollen nerve in ‘pi’ (*) with positive vascularity (orange dots), less swelling in ‘i’ and nerve compression making waist sign (down arrows). Axial view at ‘i’ (**e**,**f**): Thin Fr (arrowheads) but it is showing >2 mm bowing ((↕) calculated by making tangent between ‘s’ and ‘p’ (**e**), thick Fr (arrowheads) but it is showing <2 mm bowing (↕, distance between Fr and dotted line across ‘s’ and ‘p’) (**f**). *MN = median nerve, Fr = flexor retinaculum, CTS = carpal tunnel syndrome, s = scaphoid, p = pisiform.*

Dichotomous variables were assessed for their frequency distribution in both case and control groups. The internal honeycomb pattern was lost in the majority of cases (66.3%), but 2 (3.1%) healthy subjects also showed loss of this appearance. In order of decreasing frequencies (54.2–39.7%), other CTS parameters in cases, such as presence of vascularity, waist sign, restricted mobility and bowing of Fr > 2 mm, were also observed in a few control subjects (up to 7.7%). Fr thickness was the only consistently normal (<0.3 mm) parameter in all control patients. All of the above parameters showed statistically significant *p*-values on 2 × 2 table analysis (Table 1, Figure 4).

### 3.2. Analysis of Quantitative Data Variables

Those qualitative parameters that could be quantified were also recorded in both groups, such as thickness of Fr, bowing of Fr, and length of the entrapped nerve (waist sign) (Table 1). Bowing showed a better AUC and sn/sp than the rest. The mean values in both groups were higher in cases than controls (Table 2). An exception is the mean length of the waist, which was higher in controls (5.6 cm) than in cases (4.9 cm), although 40 cases and 5 control patients showed the waist sign. The reason for this higher value in healthy subjects was likely attributed to a smaller standard deviation (1.2 ǁ 2.0) and a higher minimum length of the waist (4.6 ǁ 2.0 mm) in control group compared to cases, respectively.

Below, all other practicable parameters of MN in CTS, assessed at different levels from DWC to 12 cm in the forearm, are described, followed by a tabulated summary of the final impression about trends of these parameters (Table 2).

### 3.3. Median Nerve Dimensions

MN dimensions were higher in cases than in controls, whether the nerve was compressed in the tunnel or away in the forearm. All CSA parameters discussed below had significant *p*-values ranging from 0.00 to 0.05.

#### 3.3.1. Cross-Sectional Area

##### Wrist

In both case and control groups, respectively, mean values of CSA at ‘pi’ (Cpi) were greater than CSA at ‘i’ (Ci), with 12.3 versus 7.2 mm^2^ and 9.2 versus 6.4 mm^2^. The maximum value of CSA was also observed at Cpi in cases, reaching up to 33 mm^2^. Unlike cases, controls showed less variation in their data. ‘pi’ proved to be a more credible site (AUC = 0.89, sn/sp = 73.5/89.2%) for diagnosing CTS than ‘i’. (Table 2).

##### Forearm

As we proceeded proximally in cases from 4 cm to 12 cm, the mean CSA of the MN decreased from 7.1 mm^2^ to 4.9 mm^2^, along with their cut-off values. The difference of mean values between case and control groups was only 0.4–0.6 mm, decreasing from distal to proximal. The case group showed higher values at all three levels in the forearm, but the variation between the two groups was minimal compared to the wrist. This could be multifactorial; in both cases and controls, the MN within the tunnel (wrist) has a smaller CSA than in the forearm, and inflammation extends less proximally from the site of compression, alongside the physiological reduction of nerve CSA proximally in the forearm.

Isolated CSA of the forearm MN did not contribute substantially to diagnosing a diseased nerve, showing poor AUC. However, the sn/sp of C12 was high at a cut-off value of 4.9 mm^2^ (82/81.5%), but with insignificant *p*-value. Hence, we recommend using the forearm site for differences and ratio calculations (Table 2).

#### 3.3.2. Transverse Length and Depth of Wrist MN

The nerve, being elliptical in shape in the wrist, had a greater transverse (Tr) dimension than its Dp. Data showed that the MN was swollen more at ‘pi’ and in the transverse direction than at ‘i’ and depth-wise. The mean, standard deviation, minimum, and maximum values were higher at ‘‘pi’ than at ‘i’ (Table 2).

#### 3.3.3. Differences (Δ) of Median Nerve CSA

##### Δ Proximal to Inlet—Forearm

In terms of Δ, the most reliable site for imaging diagnosis was the Δ between ‘pi’ and ‘12’ CSA (Cpi-C12), which had the highest AUC and sn/sp pair (0.90 and up to 89.2%). Matching values were observed at 4 and 8 cm (Table 3).

##### Δ Proximal to Inlet—Inlet (Cpi-Ci)

The AUC and sn/sp dropped to 0.74 and <67%, respectively, for this ratio, making it less reliable than Cpi-C12 (Table 3).

#### 3.3.4. Ratios of Median Nerve

##### Wrist Proximal to Inlet/Forearm

Similar to Δ, ratios also showed the most reliable site for imaging diagnosis at the ratio between ‘pi’ and 12 cm CSA (Cpi/C12), with an AUC and sn/sp pair of 0.86 and up to 81% at cut-off of 1.8 mm2. Similar values of AUC and sensitivity were observed at 4 and 8 cm (Table 4).

##### Wrist Inlet/Forearm

Ratios between ‘i’ and the forearm were less reliable and sensitive than those at ‘pi’. Here also, the most reliable measurement was at 12 cm (Ci-C12) with an AUC of 0.76. Similar values were seen at 4 and 8 cm (Table 4).

##### Proximal to Inlet/Inlet (pi/i)

Like Δ Cpi-Ci, ratios Cpi/Ci between them also did not contribute substantially to the diagnosis of CTS. Their AUC and sn/sp dropped to 0.69 and <64%, respectively, making them less reliable parameters (Table 4).

##### Flattening Ratio in Inlet (FR i) and Proximal to Inlet (FR pi)

These ratios were the least representative (AUC up to 0.46) of a diseased MN, with <54% sn/sp (Table 4).

**Figure 5:**
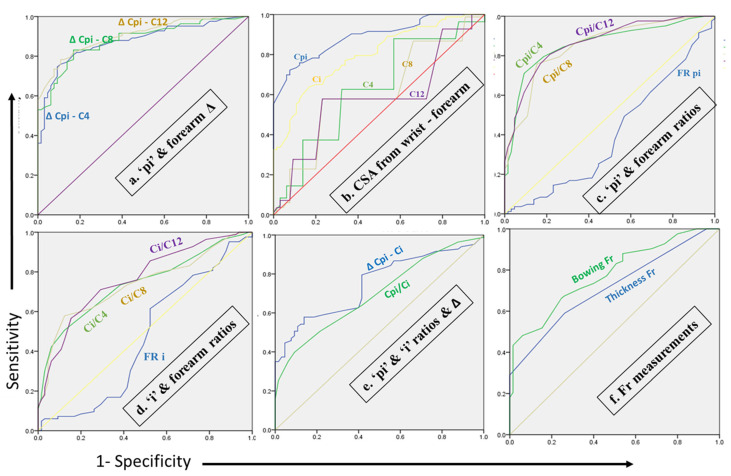
Receiver Operating Characteristic (ROC) analysis of all quantitative parameters of CTS. (**a**) Out of Δ CSA between ‘pi’ and forearm, maximum reliability was observed in Δ Cpi-C12. (**b**) Out of CSA from wrist to forearm, maximum reliability was observed at pi. (**c**) Out of ratios of CSA between ‘pi’ and forearm, maximum reliability was observed in Cpi/C12. FR ‘pi’ is unreliable and below the line. (**d**) Out of ratios of CSA between ‘i’ and forearm, maximum reliability was observed in Ci/C12. FRi is unreliable and below the line. (**e**,**f**) Ratios and Δ of ‘pi’ and ‘i’, bowing and thickness of Fr are less reliable than all other parameters. *Δ = difference, pi = proximal to inlet, i = inlet C & CSA = cross-sectional area, FR = flattening ratio, Fr = flexor retinaculum, 4,8,12 = 4,8,12 cm in forearm from distal wrist crease.*

A summary of the results of MN tracing from the carpal tunnel to the forearm up to 12 cm, along with the final impression about AUC and trend pattern of the MN, is elaborated in Table 5 and Figure 5.

## 4. Discussion

We explored the MN in wrist and forearm on ultrasound to get all its workable parameters in both healthy and CTS subjects. To the best of our knowledge, analytical, consolidated and comprehensive data of CTS under one study is not available yet. Among all observed MN measurements, the most reliable were those in which the patient’s own dimensions were used as a comparison. It included differences and ratios between CSA of MN at ‘pi’ and forearm at 12 cm, followed by CSA of MN at ‘pi’. The rest of the parameters had varying ranges of reliability, but FR was found to be the least reliable.

Carpal tunnel is an osteofibrous canal covered by the Fr and the site of nerve compression [22]. As a result of compression, the typical site of maximum edematous swelling of the MN in CTS is at the proximal edge of Fr (level of pisiform), although swelling can extend proximally and distally, as we observed [7,13,23]. Similar to our findings, the proximal tunnel outlet (just above pisiform) is also documented as a site of maximally swollen MN [7]. However, most studies consider the level of pisiform as the location of maximum MN swelling and hence its CSA [19,20,23]. Different reported mean values at the level of inlet (pisiform) ǁ proximal to inlet (just above pisiform) in cases are 12.86 ǁ 7.7 mm^2^ and 12.2 ǁ 11.0 mm^2^, which does not match our mean values of 9.2 ǁ 12.3 mm^2^. Similarly, reported values in control groups also varied, respectively: 8.5 ǁ 8.6 mm^2^ and 10.7 ǁ 10.1 mm^2^ compared to 6.4 ǁ 7.2 mm^2^ in our control hands [18,20]. The upper limit of nerve swelling is not fixed and depends on CTS severity and chronicity. The maximum observed CSA of the MN can reach up to 42.0 mm^2^ distal to the tunnel, and in our study, we observed 33 mm^2^ at proximal to tunnel inlet ‘pi’ [20]. Analogous to our method, Falsetti et al. also measured CSA just proximal to the pisiform, and their mean Cpi in cases and controls (12.1 and 5.8 mm^2^) closely matched ours (12.3 and 7.2 mm^2^) [11].

Reported cut-off values for swollen MN vary widely range between 6.5 and 15 mm^2^, with some contradictory reports of higher cut-off values at the inlet (11.1 mm^2^) than proximal to the inlet (10.5 mm^2^) [8,9,19,20,23]. Consistent with several other studies, we propose cut-off value for normal median nerve ‘Cpi’ as 9.1 mm^2^, with the best sn/sp pair of 73.5/89% [24,25]. At >10 mm^2^, specificity was 100%, but sensitivity dropped below 56%. Both Ci and Cpi were abnormal in the majority (75%) using >7.2 mm^2^ and >9.1 mm^2^ cut-offs, respectively. The remaining 25% represented either minimal or mild cases. Pisiform is the site of proximal attachment of Fr and still a part of tunnel ‘i’, so here MN has less room for swelling compared to just proximal to pisiform/inlet ‘pi’, as reflected in our data in the form of low values of ‘Ci’ compared to ‘Cpi’.

The observed inconsistencies in MN measurements may reflect a complex course of MN from hand to forearm. The MN has multifarious relationships with adjacent soft tissues at various levels of the upper limb, causing its CSA and shape to vary at each finger step along its course. This trait of MN needs to be considered seriously while taking US measurements and may explain data disparity in the literature apart from patients’ anthropometric parameters [9,22,26]. We observed a change in the shape of the MN from elliptical (within and just above the tunnel) to triangular (distal forearm) to circular (proximal forearm). Accordingly, we suggest standardizing the US methodology by defining the exact location of MN measurements with reference to US probe borders, probe line and DWC, as described in our methodology part and illustrated in Figure 2 and Figure 3.

Isolated CSA of MN in the forearm do not add significantly to the diagnosis of CTS and is usually measured to take ratios or Δ values, which is an effective way to compensate for MN variability due to physical characteristics and improve standardization of reference values [17,19,27]. In our data, AUC values for isolated forearm CSA were low (0.58–0.63) compared to ratios and Δs (0.85–0.90). Previous studies measured forearm reference points at 4, 10, or 12 cm from DWC, but not simultaneously [17,19]. We measured MN in the forearm at three equidistant points having a difference of 4 cm between them, i.e., 4, 8, 12 cm.

The best depiction of Δ was Cpi-C12, which had maximum AUC (0.90), compared to all other MN parameters. It is likely because i of the true representation of reference values, i.e., the most swollen MN at ‘pi’ and the other most normal nerve at 12 cm. Our cutoff values of Δs increased proximally from 2.2 to 3.7 mm^2^ (4 to 12 cm in forearm), keeping sensitivity close to or >80%. Cheng Xu et al. reported a threshold of 2.035 mm^2^ with 100/100% sn/sp at the proximal pronator quadratus (around 4 cm in our study) [28]. Dejaco et al. found 2.5 mm^2^ at the same level, but with lower sn/sp (93.6%/55.3%) [19].

Researchers commonly use forearm nerve reference at a distance of 4 cm or the proximal pronator quadratus, but we found the 12 cm point more suitable, as MN at 4 cm is too close to the most swollen nerve [19,27]. We calculated MN wrist-to-forearm ratios at ‘pi’ and ‘i’ in wrist versus 4, 8 and 12 cm in forearm, respectively, finding that ratios at ‘pi’ had sn/sp comparable to Δ values. Wrist to forearm ratios with ‘i’ were also significant but less representative (AUC up to 0.76) than ratios with the most swollen nerve at ‘pi’ (AUC up to 0.86). Like Δ, ratio cut-offs increased proximally from 1.3 to 1.8 (4 to 12 cm reference in forearm). Previously investigated cut-off ratios were only taken between ‘swollen nerve’ and nerve at 4 cm, 10 cm and 12 cm with 1.3, 1.4 and 1.4 ratio values, respectively. Mean values of both ratios and differences were significantly higher in cases than controls, consistent with our data [17,18,19].

Inlet to proximal to inlet CSA ratio (Ci/Cpi) or swelling ratio reflects the severity of CTS, but has insufficient sn/sp 77.9/55.5% for reliable diagnosis. Its cut-off is 1.14, but here exact locations are not specified [18]. Chang et al. took Ci/Cpi ratio between CSA of MN at pisiform level and distal radioulnar joint, corresponding to our ‘i’ and ‘pi’, respectively [29]. Instead of Ci/Cpi ratio, we evaluated Cpi/Ci ratio, recognizing that the maximum swelling of the nerve is at ‘pi’ rather than ‘i’. Our pi/i cut-off was 1.1 with low AUC and sn/sp. In a recent study, inlet (at pisiform) to proximal to inlet (just above pisiform) CSA difference (Δ Ci- Cpi) showed the highest diagnostic accuracy (cut-off 2.85 versus our 1.2) and can be used as a single best parameter to diagnose and grade CTS compared to isolated CSA values [30]. Our results for both Cpi/Ci and Δ Cpi -Ci were not very sensitive or specific to diagnose CTS (sn/sp 58.5–66.2%), but Δ Cpi -Ci had better utility (AUC = 0.74) than Cpi/Ci (AUC = 0.69).

In previous studies, FR was measured at the level of pisiform and considered abnormal if >3.0, similar to our finding [15]. Like other studies, we also found no significant (*p* = 0.49) difference between FR of case and control groups in tunnel. It also has no correlation with disease severity [29,30,31,32]. It did not add remarkably to the diagnosis of CTS (AUC = 0.46, sn/sp = 53/48%), hence making it unsuitable for diagnostic purposes. One possible explanation for it could be variable etiologies and sites of nerve compression within the tunnel.

Fr thickness and palmar bowing height increase in CTS due to adjacent MN swelling, but thicknesses vary widely between 0.6 and 3.7 mm [9,18,22,33]. Our cut-off thickness was 0.3 mm with 59% sensitivity and the mean value in case–control was 0.3–0.2 mm (*p* = 0.00). Maximum thickness was up to 0.8 mm in cases, closer to Lee et al. and Luchetti et al. [9,22]. Bowing >2 mm is commonly cited, whereas our mean was 1.4 mm (range 1.4–4.1 mm). We observed that it cannot be accurately calculated on ultrasound, so its reliability to diagnose CTS is questionable, with AUC = 0.43 versus our AUC = 0.77 with 71% sensitivity [30,34].

Swollen MN depth does not exceed >2 mm [27]. Normal people have mean Tr size of 6 mm at inlet and Dp of 2.1 mm [6,22]. In our data set, MN depth reached up to 4 mm in cases, both within and outside the tunnel. Healthy people nerve Dp was ≤2 mm and Tr dimension fluctuated between 3 and 11 mm.

MN shows movements in longitudinal and transverse directions on finger flexion–extension. US has an advantage to see these dynamics. Swollen MN shows predominantly restricted transverse movement. Its objective analysis is a tedious process without significant outcome [35,36]. We also had similar subjective observation in < 50% of cases, while asking a patient to perform pinching movement of the first two fingers. We also observed that with restricted transverse movement, the nerve tried to move in a palmar to dorsal direction, giving an impression of increasing depth Dp of the nerve. Power Doppler positivity is up to 95% sensitive and 71–100% specific in diagnosing CTS [12]. It is not always positive, which might be due to the variety of sampling data having minimal to severe cases. A less swollen nerve shows more vascularity than a severely swollen nerve [27]. We also found positive vascularity in 54.2% of cases and 4 control subjects without nerve swelling. Positive vascularity in a normal MN has a prevalence of 36%. The non-standardized US Doppler technique is a potential factor in this regard [37]. This area needs to be further studied.

**Figure 6:**
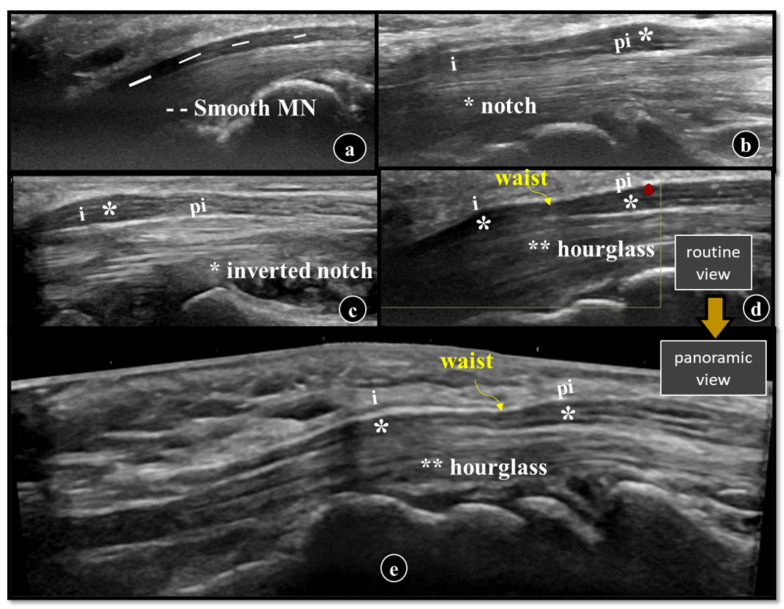
Sonographic images in longitudinal plane showing sites of swelling (*) of nerve with their corresponding signs (**a**–**e**). Normal smooth MN with no lumps and bumps (**a**). Nerve swelling in ‘pi’ giving notch sign (**b**). Nerve swelling (*) in ‘i’ or any site within inlet giving inverted notch sign (**c**). Nerve swelling (*) in both ‘pi’ and ‘i’ giving hourglass sign (combining two asterisks ** and waist) with positive vascularity at ‘pi’ (orange dot) (**d**,**e**). Routine longitudinal (**d**) and panoramic view (**e**) showing hourglass sign. US = ultrasound, DWC = distal wrist crease, MN = median nerve, pi = swollen nerve proximal to inlet, I = swollen nerve at inlet, * = swollen nerve.

The ‘waist sign’ is the compressed nerve in the inlet, while the ‘notch sign’ indicates a swollen nerve outside the tunnel. They are the ancillary imaging findings of CTS, but the direct signs of MN compression. Other terms include ‘inverted notch’ (swollen in inlet) in axial or ‘hourglass’ (swollen in both) sign in the longitudinal plane. We found either a notch or hourglass sign in our sample (Figure 6). We had positive notch signs in both cases and control groups, 48.2% and 7.7%. Kapuscinska et al. found it to be positive in 100% of cases [27]. The reason for its presence in controls can be technical factors, anatomical variability of MN, early or minimal CTS and subclinical nerve changes. These signs should be interpreted in conjunction with quantitative parameters rather than as a standalone diagnostic marker.

Honeycombing is the first subjective criteria to be noticed on US in a swollen nerve. However, the MN can look normal in 25% and 22.6% of cases, versus 33.7% in our study [7,27]. Likewise, the MN in 10% of healthy subjects can appear hypoechoic, compared with 3.1% in our data [7]. This might be due to a higher proportion of minimal or mild cases in our cohort, technical factors, or subclinical CTS.

The sonographic illustration of the MN in our prospective research at each measured point, along with the corresponding pictorial representation of the probe’s exact localization, may help standardize the methodology and avoid disparity in MN cut-off values (Figure 2 and Figure 3). Interobserver variation was not a confounding factor in our study, as sonography was performed by a single radiologist. Although we included a broad spectrum of MN parameters, this study still has some shortcomings and limitations. CTS has multifaceted aspects, and not all were covered in our study, such as the nerve–tendon ratio, ratios and Δ values with distal tunnel, and objective analysis of nerve mobility. Sub-categorization of patients according to physical features, CTS severity, duration of symptoms and etiology were not taken into account, which may influence the dimensions of MN. Anthropometric factors may influence CTS severity, nerve conduction, and consequently MN CSA values. [38]. Although covariance adjustment was not undertaken in our study, the use of intra-individual CSA ratios and differences mitigates the confounding effect of body size.

Although we studied an adequate sample size with statistically significant values, studies on a larger scale may provide a more even distribution of result data. Future research also demands work on a broader scale regarding precise specification of the inlet, proximal, and distal carpal tunnel outlet on ultrasound along with degree of reliability of values obtained at these levels for CTS diagnosis. Additionally, the presence of some positive sonographic parameters in healthy subjects in our study highlights the potential future value of US in diagnosing subclinical, impending or equivocal CTS. The aforementioned limitations emphasize the need for future stratified studies to enhance clinical applicability.

## 5. Conclusions

All qualitative and quantitative sonographic parameters in CTS were statistically significant, except for isolated CSA in the forearm and nerve flattening.

The reliability of the parameters for diagnosing CTS increases when differences and ratios are calculated, reaching maximum reliability if they are calculated between MN in wrist proximal to inlet and in the forearm at 12 cm. Their use may aid in early detection, reduce reliance on nerve conduction studies in equivocal cases, and support clinical decision making regarding conservative versus surgical management. Incorporating these parameters into routine reporting may improve diagnostic confidence and standardization.

Among isolated dimensions, CSA at proximal to inlet is the most recommended compared to other marked sites. MN flattening, the proximal to inlet-to-inlet Δ and ratio, Fr thickening and Fr bowing contribute little to the diagnosis of CTS.

## Figures and Tables

**Table 1:** (a): Table showing demographic characteristics and frequencies of all subjective MN parameters and isolated parameters of carpal tunnel syndrome (CTS) in wrist and forearm. Negative value indicates Fr below tangent. (b,c): Table showing frequencies of all subjective MN parameters and isolated parameters of carpal tunnel syndrome (CTS) in wrist and forearm. Negative value indicates Fr below tangent.

**(a) Demographic Data**						
***n* = 83~65**	**Males (n)**	**Females** **(n)**	**Age** **(years)**	**Height** **(cm)**	**Weight** **(kg)**	**BMI** **(kg/m^2^)**
**case~control**	16~19	67~46	50.6~40.16	158.3~164.7	81.2~76.21	32.3~27.9
**±** **SD**	-		8.92~9.98	7.10~8.49	14.43~16.13	5.08~4.89
***p*** **value**	-		0.00	0.00	0.03	0.00
**(b) Frequency (%) Distribution of MN and Fr Appearance (In Decreasing Order from Left to Right)**						
**Features**	Loss of Honeycomb	Positive Vascularity	Positive Waist Sign	Restricted Mobility	Bowed Fr	Thickened Fr
**case~control (%)**	66.3~3.1	54.2~6.2	48.2~7.7	43.4~6.2	39.7~1.5	28.9~0
**(n, cases)~(n, control)**	(55)~(2)	(45)~(4)	(40)~(5)	(36)~(4)	(33)~(1)	(24)~(0)
***p*** **-value**	1 × 10^−5^					
**(c) Flexor retinaculum measurements and MN compression length**						
**Features** **Mean ± SD (range)**	**Thickness of Fr (mm)** **case~control**		**Bowing height of Fr (mm)** **case~control**	**Length of waist (mm)** **case~control**		
**Mean**	0.3~0.2		2.0~1.1	4.9~5.8		
**±SD**	0.13~0.05		0.94~0.59	2.11~1.3		
**AUC**	0.71		0.77	-		
**Cut-off value**	0.3		1.4	-		
**Min.**	0.2~0.1		0.4~−0.3	2.0~4.6		
**Max.**	0.8~0.3		4.7~2.7	10.0~7.6		
***p*** **-value**	0.00			0.22		

*n = number of patients, BMI = body mass index, SD = standard deviation, Fr = flexor retinaculum, MN = median nerve, AUC = area under curve, Min = minimum, Max. = maximum.*

**Table 2:** (a–e): Table showing all isolated parameters of carpal tunnel syndrome (CTS) in wrist and forearm. Asterisks * indicates values of most reliable single parameter, out of all others mentioned, to diagnose CTS.

	AUC	Variables	Mean Case~Control	±SD Case~Control	Min. Case~Control	Max. Case~Control	Cut-Off	Sn/Sp %	*p*-Value
**(a)**	**Flexor retinaculum (mm)**								
	0.71	**Thickness**	0.3~0.2	0.13~0.05	0.2~0.1	0.8~0.3	0.3	59/73.8	0.00
	0.77	**Bowing height**	2.0~1.1	0.94~0.59	0.4~0.2	4.7~2.7	1.4	71.1/67	0.00
	-	**Waist length**	4.9~5.8	2.11~1.32	2~4.6	10~7.6	-	-	0.22
**(b)**	**Wrist median nerve CSA (mm^2^)**								
	0.80	**Inlet (Ci)**	9.2~6.4	3.32~1.43	4.5~3.4	22~9.5	7.2	72.3/71	0.00
	0.89 *	**proximal to inlet** (Cpi) *	12.3~7.2	5.57~1.57	6~3.5	33~10	9.1 *	73.5/89.2 *	
**(c)**	**Forearm median nerve CSA at (mm^2^)**								
	0.63	**4 cm (C4)**	7.1~6.5	1.61~1.50	4.5~4.1	12~11	6.9	62.7/67.7	0.01
	0.61	**8 cm (C8)**	5.8~5.4	1.19~1.27	3.2~3	9.1~10	5.9	57.8/76.9	0.05
	0.58	**12 cm (C12)**	4.9~4.5	1.18~1.05	2.7~2.1	9~8	4.9	82/81.5	0.04
**(d)**	**2D dimensions of median nerve in wrist inlet (mm)**								
	**-**	**Tr**	5.4~4.7	1.03~0.97	4.1~3	10~7	**-**	**-**	0.00
	**-**	**Dp**	1.6~1.2	0.62~0.36	1~1	4~2	**-**	**-**	
**(e)**	**2D dimensions of median nerve in wrist just** **proximal to inlet** **(mm)**								
	**-**	**Tr**	6.6~5.2	1.41~0.85	4.9~4	11~7	**-**	**-**	0.00
	**-**	**Dp**	2.2~1.6	0.51~0.32	1.2~1	3.7~2	**-**	**-**	

*AUC = area under curve, SD = standard deviation, Min = minimum, Max = maximum, Sn = sensitivity, Sp = specificity, ht = height, CSA = cross-sectional area, Tr = transverse, Dp = depth.*

**Table 3:** Table showing Δ between CSA of MN in ‘pi’ and forearm at 4, 8, 12 cm distances from distal wrist crease in both CTS cases and healthy controls. Asterisks * indicate values of most reliable single parameter, out of all others mentioned, to diagnose CTS.

	AUC	Variables (mm^2^)	Mean Case~Control	SD± Case~Control	Min. Case~Control	Max. Case~Control	Cut-Off	Sn/Sp %	*p* Value
**(a)**	**Δ proximal to inlet—forearm CSA at 4, 8, 12 cm distances from DWC**								
	0.87	**Cpi-C4**	5.2~1.3	4.68~1.08	1.4~2.5	23.9~4.9	2.2	83/83.1	0.00
	0.88	**Cpi-C8**	6.5~1.9	5.11~1.36	0.1~1.9	26.2~5.2	3.0	82/83.1	
	0.90 *	Cpi-C12 *	7.4~2.6 *	5.07~1.35	1.3~0.3	28~5.7	3.7 *	78.3/89.2 *	
**(b)**	Δ **proximal to inlet—inlet CSA**								
	0.74	**Cpi-Ci**	3.2~1.0	3.44~0.80	0~0	19~3.4	1.2	61.4/66.2	0.00

*AUC = area under curve, SD = standard deviation, Min = minimum, Max = maximum, Sn = sensitivity, Sp = specificity, ht = height, CSA = cross-sectional area, Δ = difference, pi = proximal to inlet, i = inlet.*

**Table 4:** Table showing all ratios calculated in both CTS cases and healthy controls. Overall ratios are between wrist and forearm and flatting of median nerve. Asterisks * indicate values of most reliable single parameter, out of all others mentioned, to diagnose CTS.

	AUC	Variables (mm^2^)	Mean Case~Control	SD± Case~Control	Min. Case~Control	Max. Case~Control	Cut Off	Sn/Sp %	*p* Value
**(a)**	**Wrist to forearm ratio: CSA at ‘pi’ ÷ CSA in forearm at 4, 8, 12 cm**								
	0.86	**Cpi/C4**	1.7~1.1	0.55~0.26	0.8~0.6	4.0~2.0	1.3	80/82	0.00
	0.85	**Cpi/C8**	2.1~1.4	0.78~0.36	1~0.6	5.5~2.4	1.5	80/74	
	0.86 *	Cpi/C12*	2.5~1.6	0.88~0.37	1.3~0.9	6.6~2.7	1.8 *	81/79 *	
**(b)**	**Wrist to forearm ratio: CSA at ‘i’ ÷ CSA in forearm at 4, 8, 12 cm**								
	0.74	**Ci/C4**	1.3~1.0	0.38~2.29	0.6~0.5	2.7~1.7	1.1	74/62	0.00
	0.74	**Ci/C8**	1.6~1.2	0.51~0.31	0.8~0.6	3.8~2.1	1.3	74/59	
	0.76	**Ci/C12**	1.9~1.4	0.54~0.35	1.0~0.9	3.6~2.5	1.5	71/71	
**(c)**	**Wrist ratios: CSA at ‘pi’ ÷ CSA at ‘i’**								
	0.69	**Cpi/Ci**	1.3~1.1	0.34~0.16	0.7~0.9	2.7~1.6	1.1	63.9/58.5	0.00
**(d)**	**Flattening ratio of MN at ‘i’ (Tr ÷ Dp)**								
	0.46	**FR i**	3.1~3.3	0.77~0.89	1.5~1.7	5.5~5.9	3.0	53/48	0.49
**(e)**	**Flattening ratio of MN at ‘pi’ (Tr ÷ Dp)**								
	0.40	**FR pi**	3.3~3.6	0.76~1.13	2~2.1	6.2~9.8	3.2	50/43.1	0.04

*AUC = area under curve, SD = standard deviation, Min = minimum, Max = maximum, Sn = sensitivity, Sp = specificity, ht = height, CSA = cross-sectional area, Δ = difference, pi = proximal to inlet, i = inlet, FR = flattening ratio, Tr = transverse, Dp = depth.*

**Table 5:** Tabulated summary of trends and patterns of median nerve from carpal tunnel to forearm to 12 cm.

	Overall Trend Pattern of MN Parameters from Wrist to Forearm
**(a)** **dimensions**	*Shape* of MN is elliptical in wrist and triangular to circular in forearm. *All dimensions* of median nerve in wrist (inlet or proximal to inlet) and forearm (4–12 cm) are always *more in cases* than control. *In cases and control*, *‘pi’* nerve has *higher dimension* values than ‘i’. *Proximally* from ‘*pi*’ to forearm, its *CSA decreases* and always more in cases. At *12 cm* in forearm of cases it is closest to *healthy nerve* and best to take Δ and ratios. Nerve *swells more transversely* than depth
**(b)** **differences**	Whether cases or control, Δ CSA *increases proximally* and is always *more in cases* at all levels. Δ CSA between most swollen (at proximal to inlet) and most normal nerve (at 12 cm), *pi-12* showed *best sensitivity/specificity pair and AUC*, hence most reliable to diagnose CTS Δ CSA between *pi -i is least reliable* to Dx CTS, as nerves are more swollen in wrist than forearm
**(c)** **ratios**	Whether cases or control, ratios *increase proximally* from wrist to forearm are always *more in cases.* Ratio between pi and forearm 12 cm, *pi/12* showed *best sensitivity/specificity pair, AUC* and most reliable to diagnose CTS *FR and pi/i ratio are least reliable* to Dx CTS
**(d)** **overall**	*Sensitivity/specificity/AUC of MN ratios and Δs increase* as we go *proximally* and becomes *maximum* if we choose most swollen and most normal site of MN. ΔCSA at pi-12 and isolated CSA of ‘pi’ are the *most reliable* parameters to Dx CTS. *Best CSA* of MN to Dx CTS is just proximal to pisiform rather inlet (at pisiform) with cut-off = 9.1 mm^2^ vs. 7.2 mm^2^ respectively *Best ΔCSA* to diagnose CTS is at pi-12 (cut-off = 3.7 mm^2^) *Best ratio* to diagnose CTS is CSA pi/12 (cut-off = 1.8), flattening ratio, CSA pi/i ratio, Fr thickening and Fr bowing are *not much reliable* to diagnose CTS

*MN = median nerve, pi = proximal to inlet, i = inlet, CSA = cross-sectional area, CTs = carpal tunnel syndrome, Dx = diagnose, FR = flattening ratio, Δ = difference, Fr = flexor retinaculum.*

## Data Availability

The original contributions presented in this study are included in this article. Further inquiries can be directed to the corresponding author.

## Abbreviations

The following abbreviations are used in this manuscript:

MN	Median Nerve
CTS	Carpal Tunnel Syndrome
CSA	Cross-Sectional Area
AUC	Area Under Curve
US	Ultrasound
NCS	nerve conduction study
DWC	distal wrist crease
PML	probe marker line
PUB	Probe upper border
Fr	flexor retinaculum
i	inlet
pi	proximal to inlet
Ci	Cross-Sectional Area of median nerve at inlet
Cpi	Cross-Sectional Area of median nerve at proximal to inlet
C4	Cross-Sectional Area of median nerve in forearm at 4 cm from distal wrist crease
C8	Cross-Sectional Area of median nerve in forearm at 8 cm from distal wrist crease
C12	Cross-Sectional Area of median nerve in forearm at 12 cm from distal wrist crease
Tr	transverse of median nerve
Dp	depth of median nerve
FR	flattening ratio of median nerve
Ci/C4	ratios of Cross-Sectional Area of median nerve at inlet and forearm at 4 cm from distal wrist crease
Ci/C8	ratios of Cross-Sectional Area of median nerve at inlet and forearm at 8 cm from distal wrist crease
Ci/C12	ratios of Cross-Sectional Area of median nerve at inlet and forearm at 12 cm from distal wrist crease
Cpi/C4	ratios of Cross-Sectional Area of median nerve at proximal to inlet and forearm at 4 cm from distal wrist crease
Cpi/C8	ratios of Cross-Sectional Area of median nerve at proximal to inlet and forearm at 8 cm from distal wrist crease
Cpi/C12	ratios of Cross-Sectional Area of median nerve at proximal to inlet and forearm at 12 cm from distal wrist crease
Δ	difference
Cpi-C4	difference between Cross-Sectional Area of median nerve at proximal to inlet and forearm at 4 cm from distal wrist crease
Cpi-C8	difference between Cross-Sectional Area of median nerve at proximal to inlet and forearm at 8 cm from distal wrist crease
Cpi-C12	difference between Cross-Sectional Area of median nerve at proximal to inlet and forearm at 12 cm from distal wrist crease
Sn/sp	sensitivity and specificity
